# Reduced alphabet of prebiotic amino acids optimally encodes the conformational space of diverse extant protein folds

**DOI:** 10.1186/s12862-019-1464-6

**Published:** 2019-07-30

**Authors:** Armando D. Solis

**Affiliations:** Biological Sciences Department, New York City College of Technology (City Tech), The City University of New York (CUNY), 285 Jay Street, Brooklyn, NY 11201 USA

**Keywords:** Prebiotic amino acids, Proteogenesis, Protein evolution, Protein structure, Protein backbone conformation, Residue contacts, Reduced amino acid alphabets, Mutual information, Information theory

## Abstract

**Background:**

There is wide agreement that only a subset of the twenty standard amino acids existed prebiotically in sufficient concentrations to form functional polypeptides. We ask how this subset, postulated as {A,D,E,G,I,L,P,S,T,V}, could have formed structures stable enough to found metabolic pathways. Inspired by alphabet reduction experiments, we undertook a computational analysis to measure the structural coding behavior of sequences simplified by reduced alphabets. We sought to discern characteristics of the prebiotic set that would endow it with unique properties relevant to structure, stability, and folding.

**Results:**

Drawing on a large dataset of single-domain proteins, we employed an information-theoretic measure to assess how well the prebiotic amino acid set preserves fold information against all other possible ten-amino acid sets. An extensive virtual mutagenesis procedure revealed that the prebiotic set excellently preserves sequence-dependent information regarding both backbone conformation and tertiary contact matrix of proteins. We observed that information retention is fold-class dependent: the prebiotic set sufficiently encodes the structure space of α/β and α + β folds, and to a lesser extent, of all-α and all-β folds. The prebiotic set appeared insufficient to encode the small proteins. Assessing how well the prebiotic set discriminates native vs. incorrect sequence-structure matches, we found that α/β and α + β folds exhibit more pronounced energy gaps with the prebiotic set than with nearly all alternatives.

**Conclusions:**

The prebiotic set optimally encodes local backbone structures that appear in the folded environment and near-optimally encodes the tertiary contact matrix of extant proteins. The fold-class-specific patterns observed from our structural analysis confirm the postulated timeline of fold appearance in proteogenesis derived from proteomic sequence analyses. Polypeptides arising in a prebiotic environment will likely form α/β and α + β-like folds if any at all. We infer that the progressive expansion of the alphabet allowed the increased conformational stability and functional specificity of later folds, including all-α, all-β, and small proteins. Our results suggest that prebiotic sequences are amenable to mutations that significantly lower native conformational energies and increase discrimination amidst incorrect folds. This property may have assisted the genesis of functional proto-enzymes prior to the expansion of the full amino acid alphabet.

## Background

A consensus has emerged from diverse studies, beginning with the classic Miller experiments [1], that only a subset of the canonical genetically-encoded amino acids may have existed in some abundance in the prebiotic world. Bolstered by analysis of meteorite composition [2], a reanalysis of diverse experiments simulating early conditions [3], structural and thermodynamic considerations [4, 5], and genomic and proteomic studies [6, 7], there is now wide agreement that the amino acids {A,D,E,G,I,L,P,S,T,V} were sufficiently present as life began to form, and that the polymers of these amino acids would have composed the first functional biomolecules [8]. These amino acids likely dominated the composition of polypeptides and simple proto-enzymes, whose beneficial interactions with cofactors, metals, mineral surfaces, and nucleotides may have facilitated early metabolic pathways. Biosynthetic complexity, mediated by longer, well-folded, more stable enzymes with greater specificity, is likely to have come later on [9].

The progression of metabolic evolution from simple to complex becomes plausible only if the prebiotic amino acids are themselves capable of forming early structures that are sufficiently stable to catalyze reactions and to give rise to the first metabolic pathways. There are compelling reasons to suspect that reduced alphabets can, in principle, provide the minimum ingredients for activity. Foremost, it is generally held that given any fold, not all amino acids in the sequence that can form it are critical to its structural stability and specificity [10]. Amino acids share significant similarities in physicochemical properties and also in the way they encode structure [11], as evidenced by the variety of homologous sequences that have evolved to fold into similar conformations. The hypothesis that particular folds can be formulated with a simplified amino acid alphabet is supported by experimental efforts which demonstrate that structure and function can be preserved with significant alphabet reduction [12–14]. Among these studies are successful attempts employing the prebiotic set explicitly to reduce the sequence complexity of select target proteins [15–18].

These observations lead us to ask whether there are general characteristics of the prebiotic set that endow it with unique properties relevant to structure, stability, and folding. Previous studies of the physicochemical attributes of the prebiotic set provide insight into their structural coding behavior [4, 11, 19–21]. In this work, we attempt to interrogate the uniqueness of the prebiotic set directly by comparing its structural fitness against all other possible reduced amino acid sets. Using information-theoretic analysis, we explore general structural propensities of all possible reduced sets in order to discern whether the prebiotic set distinguishes itself in the way it preserves fold information. Rather than limiting the exploration to a handful of proteins or particular folds, our work examines the reducibility of the sequence space for all extant folds inhabited by single-domain proteins. Our analysis thus spans many diverse structural families.

Because the number of folds is small compared to the diversity of protein sequences [22, 23], it has been concluded that useful folds persist once they appear in the evolutionary timeline [24]. The fold space we observe today is therefore a collection of folds that have arisen throughout evolutionary history, including some from the earliest protein sequences. Therefore, to assess the fitness of the prebiotic set in organizing ancestral folds, it makes sense, then, to investigate whether the prebiotic set is able to optimally preserve structural information in the extant fold space. Benefitting from investigations that have attempted to assign temporal order for the creation of folds [25–27], our work provides a tool to discern a probable sequence of fold evolution vis-à-vis the expansion of the amino acid alphabet from the prebiotic set of around ten to the current genetically-coded twenty.

To evaluate the fitness of a given alphabet reduction, we use an information-theoretic approach to measure the amount of structural information preserved if we expose natural protein sequence to systematic virtual mutagenesis. Related versions of this information-based methodology have proven to be effective in clustering amino acids by their structural propensities [11, 28]. Our specific goal here is to see how a particular alphabet reduction affects structural information retention in a diversity of folds, and across a wide range of sequences. In order to rigorously compare the performance of the prebiotic set to any other alternative set, we undertake an exhaustive evaluation of all possible 10-member set reductions of the amino acid alphabet space by examining the ability of each of these reductions to preserve the folds of more than 2000 single-domain proteins. This systematic computational regime allows us to thoroughly and simultaneously explore the alphabet space and fold space. An information-theoretic approach is a rigorous and effective way to explore these vast computational spaces comprehensively and efficiently, permitting a definitive evaluation of the particular structural properties of the prebiotic set of amino acids that serendipitously ushered in the first life.

Our work yields a number of results. We find that among all possible 10-letter alphabets that can be organized from the full alphabet of twenty genetically-coded amino acids, the prebiotic set appears to be optimal in forming the spectrum of local backbone structures that appear in extant proteins. The prebiotic set is also near-optimal in encoding the spectrum of single-domain folds that exist in nature currently. We observe that the ability of the prebiotic set to formulate folded conformations depends on fold class—specifically, we find strong correlation between the postulated relative temporal emergence of folds and the viability of the prebiotic set to formulate them. These results have significant implications for the early evolution of structure and function of proteins. We also learn that the prebiotic set already possesses the ability to sufficiently discriminate native folds, particularly α/β and α + β folds, from incorrect folds. This is an advantageous characteristic to have at the onset of life, since it implies that sequences formed largely by the prebiotic set were already amenable to energetically beneficial mutations that can, on aggregate, sufficiently stabilize functional conformations and facilitate efficient folding even prior to the integration of the complete 20 amino acid alphabet into the genetic code.

## Methods

In order to probe the fitness of the prebiotic amino acid set to preserve fold information, we designed a computational method to assess structural information contained in every other reduced alphabet in an efficient way. This method has two major components. First, we adopt the spirit of experimental alphabet reduction efforts by undertaking virtual mutagenesis to reduce the amino acid alphabet. Because this is a computational approach, we can do so expansively for a large, non-redundant set of single-domain proteins that span fold families, allowing us to observe general patterns regarding alphabet behavior across sequence and fold spaces. Second, we employ mutual information, an information-theoretic quantity, to evaluate the success of the extensive virtual mutagenesis in a computationally efficient Monte Carlo search across the nearly 2 × 10^15^ ways to reduce the 20-letter amino acid alphabet into a 10-letter alphabet. (This aggregate number will be derived below.) Comparing the mutual information of each of these reductions allows us to identify unique folding properties of the prebiotic set. We have applied mutual information in a wide variety of efforts to tackle questions related to protein sequence, structure, and energetics [11, 28–31]. We propose that it provides an appropriate and unbiased measure to gauge structural coding properties of reduced alphabets. The details of these two components are discussed below.

### Virtual mutagenesis

We generalize this procedure to an *n*-member reduced amino acid alphabet ${\mathfrak{R}}^n$. A simple virtual mutagenesis procedure is applied to rewrite a large non-redundant data set of single-domain protein sequences using *n* amino acids, where *n* < 20. This entails the formulation of a substitution rule $\mathcal{S}$, which prescribes how each of the 20-*n* amino acids not included in the chosen ${\mathfrak{R}}^n$ will be mutated, causing their elimination in all protein sequences in the data set.

**Fig. 1:**
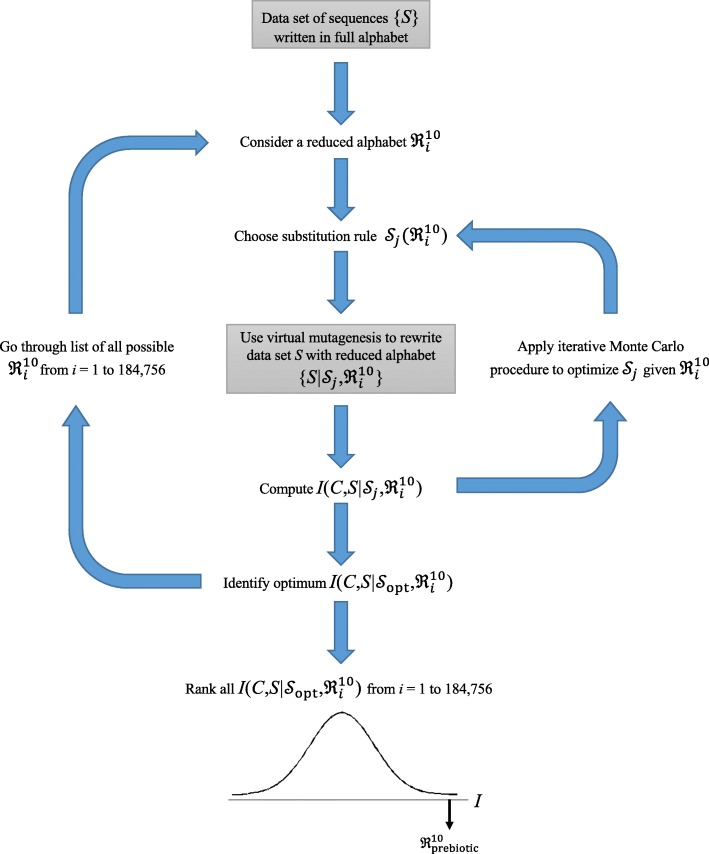
Work flow of the virtual mutagenesis procedure and mutual information optimization. A large data set of protein sequences (whose structures are known) is rewritten using a given reduced alphabet ${\mathfrak{R}}_i^{10}$, a 10-member subset of the 20 genetically coded amino acids, and ${\mathcal{S}}_j$, the substitution rule that dictates how the remaining amino acids are to be mutated virtually. For every combination of ${\mathfrak{R}}_i^{10}$ and ${\mathcal{S}}_j$, mutual information can be computed to assess their effectiveness in preserving structural information in the data set of more than 2000 single-domain proteins. Because there are more than 10^15^ different combinations of ${\mathfrak{R}}_i^{10}$ and ${\mathcal{S}}_j$ for which a mutual information can be computed, a Monte Carlo procedure is implemented to search across the different ${\mathcal{S}}_j$ efficiently given each of the 184,756 ways to configure ${\mathfrak{R}}_i^{10}$. In the end, the percentile rank of the prebiotic set ${\mathfrak{R}}_{\mathrm{prebiotic}}^{10}$ is computed from the spectrum of mutual information values given by all other alternative 10-letter alphabets

This procedure, shown graphically in Fig. 1, involves layers of recursion. Considering one particular reduced alphabet ${\mathfrak{R}}_i^n$, it is seen that there are many ways to formulate the substitution rule $\mathcal{S}$. In particular, each of the remaining (20-*n*) amino acids not part of ${\mathfrak{R}}_i^n$ can be mutated into any one of the *n* amino acids that is part of ${\mathfrak{R}}_i^n$, giving (*n*)^20*-n*^ possible $\mathcal{S}$. A computational strategy to gauge the fitness of each $\mathcal{S}$ is necessary in order to identify the optimal rule ${\mathcal{S}}_{\mathrm{opt}}$ to associate with the particular ${\mathfrak{R}}_i^n$. In our work, the optimal rule ${\mathcal{S}}_{\mathrm{opt}}$ is the best of all (*n*)^20*-n*^ possible $\mathcal{S}$ that yields the highest structural mutual information, a quantity we define below. In our investigation of the prebiotic set, for *n* = 10, there are 10^10^ unique $\mathcal{S}$ per ${\mathfrak{R}}_i^{10}$. Enumerating all 10-member sets yields 184,756 unique ${\mathfrak{R}}^{10}$. Their product illustrates the extent of the computational challenge involved—i.e., there are potentially more than 1.8 × 10^15^ virtual mutagenesis experiments to apply to each of the more than 2000 proteins in our data set.

### Evaluation of fitness of $\mathcal{S}$

We designed a measure of fitness of a particular substitution rule ${\mathcal{S}}_j$ to reflect the average viability of all amino acid substitutions across a range of conformations and sequence contexts that exist in the universe of single-domain structures. The fitness must consider the myriad local and long-range interactions that attend each residue position in the sequence.

Given the universe of protein conformations *C* and their corresponding sequences *S*, we employ the information-theoretic quantity total mutual information *I*_total_(*C*,*S|* ${\mathcal{S}}_j$, ${\mathfrak{R}}_i^n$) to measure the fitness of the reduced sequences {*S|* ${\mathcal{S}}_j$, ${\mathfrak{R}}_i^n$ } transformed by substitution rule ${\mathcal{S}}_j$ under a given alphabet reduction scheme ${\mathfrak{R}}_i^n$.

We consider the aptness of substituting one amino acid for another in terms of two general interactions: those occurring in the local sequence that dictate backbone conformations, and those occurring far in sequence that form the matrix of non-local tertiary contacts. These interactions dominate knowledge-based potential functions that perform well in protein folding investigations [32]. Similar to knowledge-based potentials [29], the total mutual information can be estimated as a sum of mutual information terms:

1 $$ {I}_{\mathrm{total}}\left(S,C|{\mathcal{S}}_j,{\mathfrak{R}}_i^n\right)={I}_{\mathrm{bb}}\left(S,{C}_{\mathrm{bb}}|{\mathcal{S}}_j,{\mathfrak{R}}_i^n\right)+{I}_{\mathrm{c}}\left(S,{C}_{\mathrm{c}}|{C}_{\mathrm{bb}},{\mathcal{S}}_j,{\mathfrak{R}}_i^n\right) $$

where *I*_bb_(*S,C*_bb_| ${\mathcal{S}}_j,{\mathfrak{R}}_i^n$) refers to the mutual information between backbone conformation *C*_bb_ and the amino acid sequence *S*; and *I*_c_(*S,C*_*c*_| ${C}_{\mathrm{bb}},{\mathcal{S}}_j,{\mathfrak{R}}_i^n$) refers to the mutual information between contacting amino acids in the tertiary fold and their Cartesian distances *C*_c_ within a specified cut-off. The dependence of *C*_c_ on *C*_bb_ (in the second term above) follows from the chain rule of probability [33].

A number of simplifications are necessary in order to make this equation workable here. Because we are using a finite data set to parameterize this equation, it is not feasible to consider the effect of the entire protein sequence *S* on structural features *C*_bb_ and *C*_c_, while also including any dependencies between them. Over-partitioning the data set to estimate probabilities, as with any knowledge-based potential, can lead to complete memorization and information degradation [29].

As a first approximation, we recognize aspects of sequence that are most relevant to the two interactions considered: for the backbone conformation *C*_bb_, the local sequence *S*_loc_ exerts the most influence [34], and for the contact conformation *C*_c_, the contacting amino acid pair *S*_c_ principally determines residue interactions [30]. Another simplification can be made if one assumes that the structural features *C*_bb_ and *C*_c_ are independent, thereby eliminating the dependence of *C*_c_ on *C*_bb_ in the second term. To take into account any overlap between *I*_bb_ and *I*_c_, one can introduce weight coefficients, a common strategy in formulating knowledge-based folding potentials to limit redundancies among potential terms [32]. Using these strategies, we can rewrite the equation as:

2 $$ {I}_{\mathrm{total}}\left(S,C|{\mathcal{S}}_j,{\mathfrak{R}}_i^n\right)\approx {I}_{\mathrm{bb}}\left({S}_{\mathrm{loc},},{C}_{\mathrm{bb}}|{\mathcal{S}}_j,{\mathfrak{R}}_i^n\right)+\alpha {I}_{\mathrm{c}}\left({S}_{\mathrm{c}},{C}_{\mathrm{c}}|{\mathcal{S}}_j,{\mathfrak{R}}_i^{n.}\right) $$

where α is a weight coefficient. In the case of independence between the two terms, α = 1. Parameterization of these two informatic terms was done using the Information Maximization Device [31] with a large non-redundant data set of high resolution structures, as described below. The weight coefficient was estimated by observing the stability of the alphabet reduction ${\mathfrak{R}}^n$ across different alphabet size *n*, described in the Results section below.

We employed this equation to estimate the mutual information for a given ${\mathfrak{R}}_i^n$ as follows. Since *S*, the reduced sequence of the proteins in the data set, will change as a result of a particular { ${\mathcal{S}}_j$, ${\mathfrak{R}}_i^n$ }, both informatic terms *I*_bb_ and *I*_c_ as well as their sum *I*_total_ will also shift. Searching across all possible substitution rules $\mathcal{S}$ identifies the best substitution rule ${\mathcal{S}}_{\mathrm{opt}}$ as that which yields the *highest* mutual information *I*_total_. We associate this *I*_total_ with the fitness of the particular ${\mathfrak{R}}_i^n$.

The fitness of the prebiotic alphabet in preserving fold information is evaluated by comparing its mutual information ${I}_{\mathrm{total}}\left(S,C|{\mathcal{S}}_{\mathrm{opt}},{\mathfrak{R}}_{\mathrm{prebiotic}}^n\right)$ with the spectra of mutual information values given by all other reduced sets, illustrated in the bottom section of Fig. 1. We also implement a gapless threading procedure (without relaxation) to approximate the ability of the simplified alphabet to discriminate natively folded conformations from incorrect sequence-structure alignments. Based on these comparisons, we are able to examine whether the prebiotic alphabet exhibits unusual properties related to structure information compared to all alternative reduced sets.

We note that the virtual mutagenesis procedure introduced here does not involve a molecular relaxation step to locally adjust the tertiary conformation in response to multiple residue substitutions. Because such a step is computationally demanding, it would not permit a comprehensive survey of the alphabet and fold spaces. But since we formulated our contact mutual information term to account for the interaction of residues within the context of their tertiary environments, any seriously destabilizing substitutions should be penalized properly. This report shows that our methodology has nonetheless been able to discern fundamental coding behavior of amino acids that would not have been evident otherwise.

### Deriving information-optimized local backbone descriptor

We designed the quantity ${I}_{\mathrm{bb}}\left({S}_{\mathrm{loc},},{C}_{\mathrm{bb}}|{\mathcal{S}}_j,{\mathfrak{R}}_i^n\right)$ to measure the influence of the local amino acid sequence on the backbone conformation given a particular reduced alphabet ${\mathfrak{R}}_i^n$ and substitution rule ${\mathcal{S}}_j$. For *S*_loc_, we considered the trimer sequence, known to carry substantial mutual information with the protein backbone [31]. For the backbone conformation, we considered the dihedral angles formed by the virtual alpha carbon bonds, which are summative descriptors of the path of the protein backbone [35, 36]. For an amino acid triplet *s*_i-1_*-s*_i_*-s*_i + 1_, we computed two dihedral angles defined along the virtual C^α^_i-1_-C^α^_i_ bond (γ_i-1_) and the virtual C^α^_i_-C^α^_i + 1_ bond (γ_i_). These two dihedral angles form a dihedral angle pair (γ_i-1_,γ_i_) that defines a space analogous to the Ramachandran space (ϕ,ψ).

Mutual information between sequence and conformation can be estimated by [31]:

3 $$ \mathrm{I}\left(S,C\right)=\sum \limits_S\sum \limits_C\ p\left(s,c\right)\ \ln \frac{p\left(c|s\right)}{p(c)}\approx \frac{1}{n_d}\sum \limits_{\begin{array}{c}\mathrm{all}\ \mathrm{str}\\ {}\mathrm{data}\end{array}}\ \ln \frac{\ f\ \left(c|s\right)}{f\ (c)} $$

where *C* stands for conformation; *c* is the particular conformation; *S* stands for sequence; *s* is the particular sequence; *p* is the probability; and *f* is the frequency, culled from the structural data set of size *n*_*d*_ amino acids. The summations in the middle equation cover all instances of *S* and *C* in the protein universe, while the summation in the right-hand side covers all instances of *S* and *C* in the representative, non-redundant structural data. In order to ensure that each data point does not influence its own contribution to the mutual information, a condition that becomes critical as the number of discrete partitions of *f* increases [28], the frequencies *f* are subtracted by 1 (i.e., removing itself from the frequency estimates). In addition, where appropriate, estimates for *f* are buttressed by background frequencies to guard against complete memorization [31].

Applying this mutual information equation to estimate backbone information contained in the trimer sequence *s*_*i*-1_-*s*_*i*_-*s*_*i* + 1_, given a particular alphabet ${\mathfrak{R}}_i^n$ and substitution rule ${\mathcal{S}}_j$, we have:

4 $$ {I}_{\mathrm{bb}}\left({S}_{\mathrm{loc},},{C}_{\mathrm{bb}}|{\mathcal{S}}_j,{\mathfrak{R}}_i^n\right)\approx \frac{1}{n_d}\sum \limits_{\begin{array}{c}\mathrm{all}\ \mathrm{str}\\ {}\mathrm{data}\end{array}}\ \ln \frac{\ f\ \left({\gamma}_{i-1},{\gamma}_i|{s}_{i-1}-{s}_i-{s}_{i+1}\right)}{f\ \left({\gamma}_{i-1},{\gamma}_i\right)} $$

It is necessary to discretize the (γ_i-1_,γ_i_) space due to the paucity of high quality structural data. The method used to determine the information-optimal partition of the (γ_i-1_,γ_i_) space is based on the Voronoi partition [37]. The Voronoi partition can be established by choosing *k* seeds (points in the space) to define *k* Voronoi cells, within which every point is closest to the seed that defines the cell. The goal is to maximize the effect of local sequence on structure—i.e., to compare the information entropies of sequence-independent and sequence-dependent propensities and subsequently identify the Voronoi partition of the space that maximizes this difference.

A Monte Carlo algorithm was applied to identify the optimal set of *k* seeds {*v*_1_,*v*_2_, …,*v*_k_} needed to partition the space, where *v*_i_ is a point in the structural space. We used mutual information as the objective function:

5 $$ {I}_{\mathrm{V}}\left({S}_{\mathrm{loc},},{C}_{\mathrm{bb}}|{V}^k\right)\approx \frac{1}{n_d}\sum \limits_{\begin{array}{c}\mathrm{all}\ \mathrm{str}\\ {}\mathrm{data}\end{array}}\ \ln \frac{\ f\ \left({V}^k\left({\gamma}_{i-1},{\gamma}_i\right)|{s}_i\right)}{f\ \left({V}^k\left({\gamma}_{i-1},{\gamma}_i\right)\right)} $$

where *V*^*k*^(*γ*_*i* − 1_, *γ*_*i*_) is the Voronoi cell to which the dihedral angle pair (γ_i-1_,γ_i_) belongs. To simplify this optimization, we restrict the sequence effect to the central amino acid in position *i* (instead of considering the full trimer sequence). To facilitate the search, the (γ_i-1_,γ_i_) space was discretized to 0.1 radians, covering the space by 62 × 62 or 3844 equidistant points.

A Monte Carlo run begins with randomly selecting *k* initial seeds out of the 3844 points and then assigning all the (γ_i-1_,γ_i_) angle pairs in the structural data set to the closest seed in the initial set using Euclidean distance. With each (γ_i-1_,γ_i_) pair assigned to its Voronoi cell, the mutual information between sequence and structure can be computed according to Eq. (5). The Monte Carlo search commences by generating an alternative set of seeds and recomputing the mutual information to test whether to accept the new set of seeds. Two ways of generating alternative seeds were used: one, by randomly replacing 1, 2, or 3 seeds (or more, depending on the total number of seeds) by other possible seeds picked randomly from the 3844 points; and two, by randomly moving 1, 2, or 3 seeds (or more) to nearby points in the grid.

This algorithm yields ${V}_{\mathrm{opt}}^k$, the optimal discretization of the (γ_i-1_,γ_i_) space into *k* points, by maximizing the mutual information across different sets of seeds {*v*_1_,*v*_2_, …,*v*_k_} sampled in the Monte Carlo search:

6 $$ {V}_{\mathrm{opt}}^k=\overset{\arg \max }{\left\{{v}_1,{v}_2,\dots, {v}_k\right\}}\ {I}_{\mathrm{V}}\left({S}_{\mathrm{loc},},{C}_{\mathrm{bb}}|{V}^k\right) $$

**Fig. 2:**
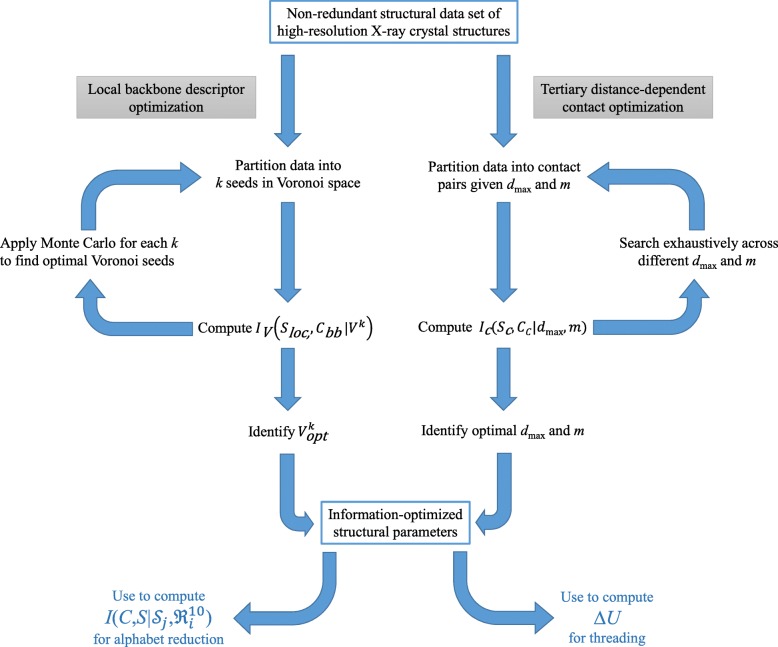
Work flow of the structural descriptor optimization used to parameterize mutual information. The backbone structure is characterized by a pair of virtual alpha carbon dihedral angles, whose two-dimensional space can be discretized by the Voronoi partition into *k* states or seeds. The number of seeds *k* and their locations in the dihedral angle space can be optimized by a Monte Carlo search using mutual information as objective function, as illustrated on the left side of the Figure. The tertiary contact structure is characterized by the contact distances between pairs of non-adjacent residues, with the parameters *d*_max_ that describe the maximum distance of interaction and *m* that dictates the number of discrete bins by which the length *d*_max_ is partitioned. An exhaustive search is made across various *d*_max_ and *m* with mutual information as objective function. The two sets of information-optimized descriptors (for the backbone and for tertiary contacts) are used to compute the mutual information *I*_bb_ and *I*_total_ used in the virtual mutagenesis procedure (see Fig. 1), and also used to parameterize the energy function Δ*U* employed in the threading experiment

This procedure also identifies the optimal *k*, the number of cells, since the Information Maximization Device [31] assigns low mutual information to extreme values of *k*. Specifically, for low *k*, mutual information is low because the resolution is too coarse to capture nuances of structural information in sequence; while for high *k*, mutual information is also low because the size of the structural data set becomes insufficient to populate frequency distributions in any meaningful way. This optimization procedure is illustrated in the left half of Fig. 2.

Representing the assignment of backbone conformation into the optimal Voronoi cells as ${V}_{\mathrm{opt}}^k\left({\gamma}_{i-1},{\gamma}_i\right)$, we can rewrite the backbone term as:

7 $$ {I}_{\mathrm{bb}}\left({S}_{\mathrm{loc},},{C}_{\mathrm{bb}}|{\mathcal{S}}_j,{\mathfrak{R}}_i^n\right)\approx \frac{1}{n_d}\sum \limits_{\begin{array}{c}\mathrm{all}\ \mathrm{str}\\ {}\mathrm{data}\end{array}}\ \ln \frac{\ f\ \left({V}_{\mathrm{opt}}^k\left({\gamma}_{i-1},{\gamma}_i\right)|{s}_{i-1}-{s}_i-{s}_{i+1}\right)}{f\ \left({\gamma}_{i-1},{\gamma}_i\right)} $$

Another kind of backbone structure mutual information was computed in order to account for uneven frequencies of amino acids in the data set. In contrast to the quantity *I*_bb_ above, which considers the unweighted triplet contribution to the mutual information, we formulate *I*_bb,norm_ to represent the triplet-sequence normalized case in which each of the 8000 triplets contribute equally to the mutual information:

8 $$ {I}_{\mathrm{bb},\operatorname{norm}}\left({S}_{\mathrm{loc},},{C}_{\mathrm{bb}}|{\mathcal{S}}_j,{\mathfrak{R}}_i^n\right)\approx \frac{1}{8000}\sum \limits_{\begin{array}{c}\mathrm{all}\\ {}\mathrm{trimers}\end{array}}\ \frac{1}{n_{s_{i-1}-{s}_i-{s}_{i+1}}}\sum \limits_{s_{i-1}-{s}_i-{s}_{i+1}}\ \ln \frac{\ f\ \left(V\left({\gamma}_{i-1},{\gamma}_i\right)|{s}_{i-1}-{s}_i-{s}_{i+1}\right)}{f\ \left({\gamma}_{i-1},{\gamma}_i\right)} $$

where the inner summation is done for all occurrences of the particular trimer sequence *s*_*i* − 1_ − *s*_*i*_ − *s*_*i* + 1_, with ${n}_{s_{i-1}-{s}_i-{s}_{i+1}}$ as the number of occurrences; and the outer summation goes through all 8000 unique trimers in the data set. This supplemental quantity was computed to check whether more frequently occurring amino acids can bias the measure. This normalization adjustment removes the bias of amino acid frequency so that all 20 amino acids become equally likely to formulate functional structures. This is certainly not how polypeptides behaved in the prebiotic world, but we undertake this analysis to see how well the prebiotic set can sufficiently cover the *sequence-normalized* structural space that is available to all local sequences regardless of relative frequency. (Since it is not possible to normalize the amino acid composition for *I*_total_ because of the cooperative nature of the contact environment, no adjustments were done to limit composition effect).

### Deriving information-optimized non-local distance-dependent descriptor

Each residue in the folded protein interacts directly with its molecular environment. The object of the term ${I}_{\mathrm{c}}\left({S}_{\mathrm{c}},{C}_{\mathrm{c}}|{\mathcal{S}}_j,{\mathfrak{R}}_i^n\right)$ is to quantify the structural information encoded in the contact environment of every residue in the context of the tertiary structure, which allows us to assess the ability of any reduced alphabet to preserve this information. Mutual information is measured on a per residue basis, computed as the mean sum of the information contribution of all individual contacts within a cut-off distance:

9 $$ {I}_{\mathrm{c}}\left({S}_{\mathrm{c}},{C}_{\mathrm{c}}|{\mathcal{S}}_j,{\mathfrak{R}}_i^n\right)\approx \frac{1}{n_d}\sum \limits_{\begin{array}{c}\mathrm{all}\ \mathrm{str}\\ {}\mathrm{data}\end{array}}\ \sum \limits_{\begin{array}{c}\mathrm{all}\ \mathrm{contacts}\\ {}\mathrm{for}\ \mathrm{residue}\ \mathrm{i}\\ {}\mathrm{within}\ {d}_{\mathrm{max}}\end{array}}\ln \frac{\ f\ \left({d}_c|{s}_i{s}_j\right)}{f\ \left({d}_c\right)} $$

where *d*_*c*_ is the distance (nearest atom approach) between two amino acid residues *s*_*i*_ and *s*_*j*_ at least five residues apart in sequence, with a maximum distance *d*_max_, capped up to 12 Å, around the effective upper range for long-range interactions [11].

Because there are a range of *d*_max_ values, and because *d*_*c*_ can be discretized at different resolutions, the Information Maximization Device [31] can be applied to these two parameters. Using the equation above, setting ${\mathfrak{R}}^{20}$ (for the full 20-letter alphabet), and using unreduced sequences, we tested a range of values [4.0 Å,12.0 Å] for *d*_max_ as well as different levels of discretization *m* within the range [4100], in order to identify the optimal parameters automatically. As in previous work [30], the optimum discretization level is expected to be fine enough to permit discrimination but not so fine that the limited data set would not be able to support it. The Information Maximization Device [31] assures that this optimum level will be found. This optimization procedure is illustrated in the right half of Fig. 2.

### Monte Carlo procedure to optimize prescriptive alphabet reduction via virtual mutagenesis

Given a particular reduced alphabet ${\mathfrak{R}}_i^n$, finding the most optimal substitution rule ${\mathcal{S}}_{\mathrm{opt}}$—that which gives the highest mutual information—may be done by an exhaustive enumeration and evaluation of all 10^10^ unique ways to configure $\mathcal{S}$. However, this becomes cumbersome because the virtual mutagenesis procedure involves rewriting the sequences of all the proteins in a data set of 2141 structures and then recalculating the mutual information for each unique $\mathcal{S}$. An alternative is to apply another Monte Carlo search, using mutual information as objective function, to identify ${\mathcal{S}}_{\mathrm{opt}}$, a reasonable approach for search spaces that are particularly smooth. This approach avoids having to evaluate all 10^10^ unique states of $\mathcal{S}$.

The computational procedure, shown in Fig. 1, is as follows: (1) a virtual mutagenesis of protein sequences in the structural data set is made globally to reduce the alphabet to the desired level ${\mathfrak{R}}_i^n$ by using an initial, randomly chosen substitution rule ${\mathcal{S}}_{\mathrm{initial}}$; (2) the mutual information is computed; (3) the substitution rule is slightly altered (by reassigning a random number of amino acids from one member of the reduced alphabet to another), the virtual mutagenesis is reapplied following the new rule $\mathcal{S}^{\prime }$, and the mutual information is recalculated; (4) the new substitution rule $\mathcal{S}^{\prime }$ is accepted if the mutual resulting information is lower, or discarded otherwise; (5) steps 3–4 are repeated until the mutual information does not decrease any further for 10,000 iterations, resulting in ${\mathcal{S}}_{\mathrm{local}\ \max }$; (6) steps 1–5 are repeated 20 times; (7) the lowest mutual information among the 20 maxima ${\mathcal{S}}_{\mathrm{local}\ \max }$ identifies the optimal substitution rule for the given reduced alphabet. Frequently, we find that more than one of the 20 maxima ${\mathcal{S}}_{\mathrm{local}\ \max }$ gives ${\mathcal{S}}_{\mathrm{opt}}$. Comparing results of the exhaustive enumeration with this Monte Carlo procedure for 100 randomly chosen reduced alphabets, we observe that all of the ${\mathcal{S}}_{\mathrm{opt}}$ was identified by the latter, while requiring only 10^6^ instead of 10^10^ iterations. This computational shortcut becomes especially beneficial because optimization for ${\mathcal{S}}_{\mathrm{opt}}$ has to be done for all possible 184,756 reduced alphabets.

### Randomized sequence threading to assess the ability of reduced sequences to preserve fold information

Gapless threading without relaxation using a knowledge-based potential was done to probe the ability of the informatic measurements for reduced amino acid sets to discriminate native from incorrect sequence-structure alignments. This simulation was done to evaluate the potential for reduced sequences to be optimized for a crucial characteristic of evolved soluble proteins: a pronounced gap between the energy of the native fold and the mean energy of incorrect folds. Such energy landscapes depict a folding process that occurs with relative ease and that results in native conformations with pronounced stability mediated by an aggregate of beneficial interactions that act with “minimum frustration” [38]. It is of interest to know whether this property was already operational early in the evolution of the first functional polypeptides.

In order to neutralize amino acid composition effects while retaining the ability to do fold recognition regardless of sequence length, the strategy adopted here was to randomly shuffle protein sequences and then remount them onto the original structure [39, 40]. This has been shown to produce statistics very similar to the more traditional approach to threading (by mounting a sequence onto different folds) while reducing computing time [32]. Because this sequence shuffling simulation is being done to 2141 single-domain proteins multiple times, for each of the 184,756 different sets of 10 amino acids, relaxation of the threaded conformations is not feasible. It is assumed, however, that a general approximation to the sequence-structure alignment is still informative because of the vastness of the sequence-structure space that we are permitted to explore with this approximation.

The potentials used to gauge threading fit are derived by recasting propensities used in mutual information measurements as empirical energies using the Boltzmann formalism [29, 41]. The resulting potentials of mean force, derived from the Boltzmann equation relating propensities to free energies, give a first approximation of the energetics of folding. To summarize, the Boltzmann formalism relates observed probabilities associated with conformation *C* and sequence *S* to the potential Δ*U*:

10 $$ \Delta  U=- kT\ln \frac{p\left(C|S\right)}{p(C)} $$

where *k* is the Boltzmann constant and *T* is the absolute temperature. If we consider the backbone potential and the contact potential as components of the total potential, the equation becomes:

11 $$ \Delta  U=\Delta  {U}_{bb}+\Delta  {U}_c=- kT\ln \frac{p\left({C}_{bb}|{S}_{\mathrm{loc}}\right)}{p\left({C}_{bb}\right)}- kT\ln \frac{p\left({C}_c|{S}_c\right)}{p\left({C}_c\right)} $$

Applying this to discrete instances of backbone states *c*_*bb*_ and contacts *c*_*c*_ in a given protein, the equation becomes:

12 $$ \Delta  U=- kT\sum \limits_{\begin{array}{c}\mathrm{all}\\ {}\mathrm{residues}\end{array}}\ln \frac{p\left({c}_{bb}|{s}_{\mathrm{loc}}\right)}{p\left({c}_{bb}\right)}- kT\sum \limits_{\begin{array}{c}\mathrm{all}\\ {}\mathrm{residues}\end{array}}\sum \limits_{\begin{array}{c}\mathrm{all}\\ {}\mathrm{contacts}\end{array}}\ln \frac{p\left({c}_c|{s}_i{s}_j\right)}{p\left({c}_c\right)} $$

The two component terms above are parameterized the same way as Eqs. (7) and (8), estimating probability distributions as frequency distributions derived from the same non-redundant data set of protein structures.

The equation above can be used to situate the energy score of the native sequence-structure alignment, Δ*U*_prebiotic_, in a spectrum of scores given by repeated threading of shuffled sequences onto their respective conformations. We found that 500 sets of shuffled sequences provided a converging estimate of the difference between Δ*U*_prebiotic_ and < Δ*U*_shuffled_>, the mean energy of incorrect alignments. This difference measures the relative stability of the native conformation against alternative (incorrect) conformations. With repeated measurements across different reduced alphabets, the ability of the prebiotic alphabet to preserve this property can be assessed.

In the end, discrimination is measured by total divergence, the difference between the mutual information of the native-sequence-structure alignment and the mean divergence, a quantity derived from the average energy scores of 500 shuffled sequence-structure alignments. Total divergence works as well as the more familiar *Z*-score in measuring the effectiveness of potential functions in identifying native sequence-structure alignments [29].

### Structural data sets

A data set of 4641 non-redundant high-resolution X-ray crystal structures was culled from PISCES [42] with pairwise sequence identity of no greater than 25%, resolution of at least 2.0 Å, and R-factor cutoff at 0.25. This set was used to optimize the parameters for the structural descriptors in the mutual information equations (Eqs. 7–9) and the threading potential (Eq. 12), and also to derive the structural propensities *f* in these equations. The Information Maximization Device [31] was applied to achieve the highest resolution possible for the backbone and contact structural descriptors given this data set.

For the survey of fold and alphabet spaces, we restricted the virtual mutagenesis simulation to the subset composed of single-domain proteins, numbering 2141 high resolution structures. The SCOP database [43] was used to classify these single-domain structures into fold classes.

## Results

We examined the performance of all alphabet reductions ${\mathfrak{R}}^n$ in preserving fold information across all single domain folds, in order to explore the viability of alphabet reduction across fold space. We note that the alphabet reduction patterns detected in this work are global properties, coming from averaged structural propensities exhibited by the reduced sets across a variety of protein sequences from different fold families and of diverse functions. To accomplish the exploration across reduced alphabet space and fold space, we employed virtual mutagenesis (Fig. 1) using an information-theoretic objective function, the mutual information (Eq. (2), designed to embody local sequence effects on the backbone and long-range sequence effects on side chain contacts, two principal sequence-dependent interactions in folded proteins.

We also examined the case where alphabet reduction was done under a revised objective function containing only local sequence effects on the protein backbone (Eqs. 7 and 8), in order to test particular hypotheses on the emergence of early proto-enzymes in the prebiotic world. Finally, we applied a fold recognition (threading) procedure to assess the ability of reduced alphabets to retain the discriminatory power of the full alphabet in determining the native fold.

We defined structural information as a combination of two sequence-dependent interactions: that of the local backbone and that of long-range residue pair contacts. To capture the greatest possible mutual information given limited structural data, we simplified the structural descriptors into discrete states, and chose the best discretization by an optimization for mutual information, using the Information Maximization Device [31]. We then used these optimized descriptors to parameterize the mutual information equations in subsequent alphabet reduction procedures.

### Optimizing sequence-dependent local backbone and long-range contact information

**Fig. 3:**
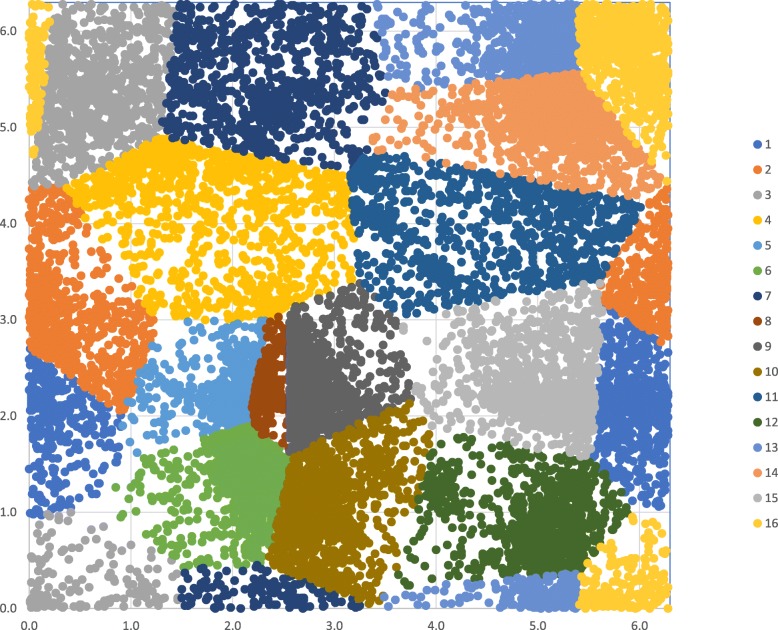
The optimal Voronoi tessellation of the virtual alpha carbon dihedral angle pair. The optimal number of polyhedra was found to be 16, and the seeds for each of these are specified in Table 1. This figure includes only 1000 random data points for each of the 16 polyhedra for illustration purposes only. (Significantly more data points are contained in the structural data set used to optimize this space)

We considered the effect of the trimer sequence (*s*_*i*-1_-*s*_*i*_-*s*_*i* + 1_) on the dihedral angle γ_i-1_ of the virtual alpha carbon bond between (*s*_*i*-1_-*s*_*i*_) and the dihedral angle γ_i_ of the virtual alpha carbon bond between (*s*_*i*_-*s*_*i* + 1_). A Monte Carlo optimization (Fig. 2) using mutual information as the objective function was applied to different levels of partition of the space, represented by *k,* from *k* = 8 to *k* = 25. We found that *k* = 16 yielded the highest mutual information. This optimal *k* was subsequently chosen to represent backbone structure in this study. The effective outcome of this optimization is the partition of the (γ_i-1_,γ_i_) space into Voronoi polyhedra specified by the seeds enumerated in Table 1. Figure 3 illustrates this optimal tessellation, which we subsequently used to parameterize the local backbone interactions, as expressed in Eq. (7).

**Table 1:** Optimal seeds for Voronoi tessellation of backbone dihedral angle pair

seed #	γ_i-1_	γ_i_
1	0.0	2.4
2	0.3	2.8
3	0.6	5.9
4	1.8	3.8
5	1.9	2.2
6	2.1	1.5
7	2.2	5.7
8	2.5	2.1
9	2.6	2.1
10	3.0	1.3
11	4.6	4.0
12	4.6	0.9
13	4.7	6.0
14	4.8	5.0
15	4.9	2.5
16	6.1	6.0

The variables γ_i-1_ and γ_i_ characterize the dihedral angles (in radians) operating on the virtual C^α^_i-1_-C^α^_i_ and C^α^_i_-C^α^_i + 1_ bonds respectively. These seeds define the information-optimal Voronoi tessellation of the virtual dihedral angle space into 16 polyhedra (illustrated in Fig. 3). Each point in the two-dimensional space can be assigned to one of the 16 partitions to which seed it is closest, based on Euclidean distance

**Fig. 4:**
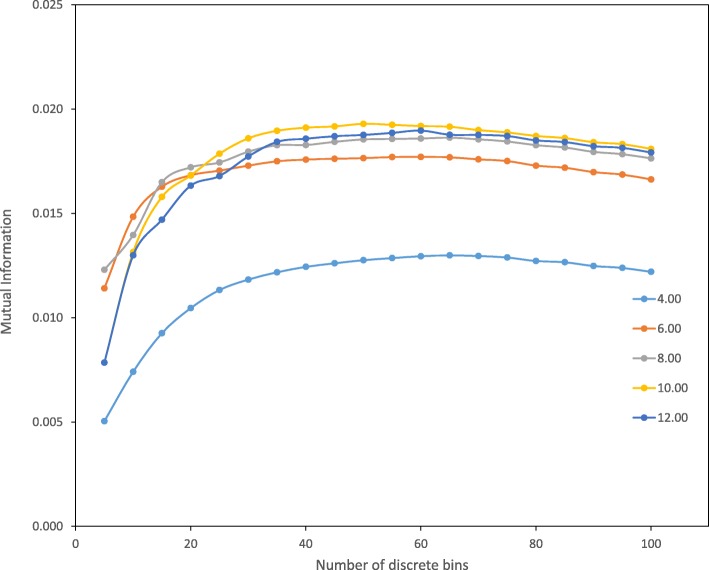
Mutual information measurements for different cut-off distances (Å) and different numbers of bins made to characterize the distance-dependent contact interaction. The optimum was found to be *d*_max_ = 10.00 Å, and the number of bins *m* = 50

The descriptor used for the contact mutual information is the distance-dependent contacts between residue pairs within *d*_max_ at nearest atom approach, which aims to encapsulate the intramolecular environment of each residue in the folded protein. An exhaustive procedure (Fig. 2) was implemented to find the most informative value for *d*_max_ as well as the number of discrete bins *m* that subdivide the contact distance. Figure 4 shows the mutual information arising from different values of *d*_max_ and discretization levels *m*. We find that the mutual information is maximal when *d*_max_ = 10 Å and *m* = 50, and used these values to parameterize the distance-dependent interactions as expressed in Eq. (9).

### Combining local and long-range sequence-dependent structural information

Due to the cooperative nature of protein folding, overlaps exist in the mutual information contained in local backbone structure and in long-range contact environments of residues. It is difficult to disentangle this overlap or to consider a grand mutual information equation encompassing both interaction domains due to the multivariate nature of such an equation vis-à-vis the limited size of the structural database with which to parameterize it. A common solution is to do a weighted sum of the two terms, as in Eq. ((2), where α is the weight coefficient. To determine an appropriate range of α, we examined the stability of the reduced alphabets across different weights, to see how dependent our results are on such weights. We observe that the optimized reduced alphabets are remarkably stable; they do not change across a wide range of weights, defined by α = [0.05,20.0] (effectively setting relative ratios up to a factor of 400), and only at extreme values of α does the influence of the over-weighted interaction become dominant. That is, near α = 0, local backbone propensities of the amino acid sequence dominate the patterns observed in the alphabet reduction procedure; conversely, at α >> 20, long range propensities dominate. This observation accords with other observations of the high consistency of amino acids in coding both local and long-range interactions [11]. To simplify our computation, we selected the neutral weighting, at α = 1, to equalize the structural information contribution of each term. This weight coefficient has been found to also be optimal in a rigorous study of knowledge-based potential functions [32].

### Optimal reduced alphabets and structural information measurements for all possible alphabet reductions

We considered two forms of the information-theoretic objective function: one that considers only the effect of the local sequence on backbone structure, *I*_bb_ (Eq. 7), and another that considers as well a non-local interaction between residues occurring in close proximity in the folded protein, *I*_total_ (Eq. (2). The former simulates prebiotic conditions where short polypeptides aggregated non-specifically with other cofactors, nucleotides, and organic molecules without well-defined tertiary folds. In such conditions, the primary determinant of structural propensity for these earliest polypeptides with nascent functions must have been the local sequence. Considering only the local sequence effect on backbone structure under a crowded non-specific environment, as expressed in *I*_bb_ (sequence-unweighted, Eq. 7) and *I*_bb,norm_ (triplet-sequence normalized, Eq. 8) thus measures the fitness of a reduced alphabet in such early conditions. In contrast, incorporating specific intra-sequence non-local interactions between residues into the objective function, as expressed in *I*_total_, permits an evaluation of the fitness of reduced alphabets in preserving fold information in the context of the folded environment.

Using a Monte Carlo search, we identified ${\mathcal{S}}_{\mathrm{opt}}$ for each of the 184,756 unique ${\mathfrak{R}}_i^{10}$, for the *I*_bb_, *I*_bb,norm_, and *I*_total_ objective functions, and then ranked them with respect to these informatic quantities. The optimal substitution rules ${\mathcal{S}}_{\mathrm{opt}}$ for the reduced alphabets ${\mathfrak{R}}_{\mathrm{best}}^{10}$ for five SCOP classes examined in this work are summarized in Table 2. The optimal substitution rules for the prebiotic set are also included in the table. Noting that these substitution rules represent averaged behavior across different folds within the major fold classes, we make a number of observations. The substitution rules and the best reduced alphabets appear to be dependent on fold class. The prebiotic set overlaps significantly with the optimal reduced alphabet for α/β and α + β folds for *I*_bb_; specifically, they differ only on one amino acid, where I is displaced by K. For the other folds, the numbers of differing amino acids are greater: two for small proteins (IL displaced by CK), and three for all-α (ITV displaced by KNQ) as well as for all-β (AIL displaced by HKN). A similar pattern emerges for the optimization on *I*_total_. The number of differing amino acids is minimal for α/β and α + β folds: for the α/β fold class, I and T are displaced by K and Y; and for the α + β fold class, I and S are displaced by R and Y. The numbers of differing amino acids for the other folds are again greater: three for all-α (IST displaced by CQY), four for small proteins (EILT displaced by CFRY), and five for all-β (ADEIL displaced by CFNRY). The substitution rules reflect expected patterns based on polarity: hydrophobic amino acids are recruited to replace other hydrophobic amino acids, and polar/charged amino acids likewise replace other polar/charged amino acids. These substitution patterns are consistent with earlier findings on structure-based amino acid clustering [11].

**Table 2:** Optimal and prebiotic reduced alphabets and substitution rules for different SCOP fold classes

Interaction domain	Fold class [SCOP #]	${\mathbf{\mathcal{S}}}_{\mathrm{opt}}\left({\mathfrak{R}}_{\mathrm{best}}^{\mathbf{10}}\right)$ ACDEFGHIKLMNPQRSTVWY	${\mathbf{\mathcal{S}}}_{\mathrm{opt}}\left({\mathfrak{R}}_{\mathrm{prebiotic}}^{\mathbf{10}}\right)$ ACDEFGHIKLMNPQRSTVWY
backbone (*I*_bb_)	all-α [1]	**A**A**DE**L**G**QL**KL**A**NPQ**A**S**KLAL	**A**A**DE**A**G**E**I**E**L**ED**P**AA**STV**AA
	all-β [2]	TV**DE**V**GH**V**K**VV**NP**TT**STV**VV	**A**T**DE**V**G**T**I**T**L**ID**P**TT**STV**VV
	α/β [3]	**A**L**DE**L**G**SV**KL**LD**P**EK**STV**AL	**A**V**DE**L**G**D**I**E**L**ID**P**EE**STV**LL
	α + β [4]	**A**V**DE**V**G**TV**KL**LD**P**EK**STV**LV	**A**V**DE**I**G**T**I**E**L**LD**P**ET**STV**IV
	small [7]	**ACDE**C**G**SV**K**VKD**P**ST**STV**VV	**A**S**DE**T**G**S**I**T**L**LD**P**TT**STV**II
backbone + contact (*I*_total_)	all-α [1]	**ACDE**L**G**QLE**L**AD**PQ**QEEL**WY**	**A**A**DE**I**G**S**I**E**L**LD**P**EE**STV**LA
	all-β [2]	T**C**NS**FG**TVTVV**NP**T**RSTV**Y**Y**	**A**V**DE**V**G**T**I**S**L**VS**P**TT**STV**IV
	α/β [3]	**A**V**DE**L**G**SV**KL**YD**P**KK**S**S**V**Y**Y**	**A**V**DE**I**G**S**I**E**L**LD**P**ES**STV**IT
	α + β [4]	**A**V**DE**L**G**TVR**L**VD**P**R**R**T**TV**Y**Y**	**A**V**DE**I**G**T**I**T**L**ID**P**ST**STV**IT
	small [7]	**ACD**D**FG**YVRFFS**P**S**RS**S**V**Y**Y**	**A**V**DE**V**G**S**I**T**L**IS**P**TT**STV**TT

${\mathcal{S}}_{\mathrm{opt}}\left({\mathfrak{R}}_{\mathrm{best}}^{10}\right)$ refers to the optimal substitution rule for the reduced alphabet with the highest mutual information; ${\mathcal{S}}_{\mathrm{opt}}\left({\mathfrak{R}}_{\mathrm{prebiotic}}^{10}\right)$ refers to the optimal substitution rule for the prebiotic reduced alphabet. The amino acids are indicated in alphabetical order by their single-letter code. The amino acids in bold are included in the reduced alphabet, and those not in bold are the amino acids that substitute for those not in the reduced alphabet. Two interaction domains are considered: *I*_bb_ refers to the mutual information between local trimer sequence and alpha-carbon virtual dihedral backbone; *I*_total_ refers to the total mutual information that includes *I*_bb_ plus the mutual information arising from contacting residues in tertiary structure

To assess the effectiveness of ${\mathfrak{R}}_{\mathrm{prebiotic}}^n$ in preserving fold information compared to the spectrum of alternative reduced alphabets, we counted the number of alphabets (out of 184,756) that have *I*_bb_, *I*_bb,norm_, and *I*_total_ values higher than those of the prebiotic set. Results, summarized in Table 3, show that sequences formed from the prebiotic set preserve backbone information across all folds better than most any other reduced alphabet—i.e., ranking 48th out of 184,756 alphabets (at 99.97 percentile) for the unweighted measure *I*_bb_, and 859th (at 99.54 percentile) for the normalized measure *I*_bb,norm_. This points to the ability of the prebiotic set to completely cover the backbone structure space of extant functional folds. The optimality of the prebiotic set is somewhat reduced when the tertiary structure environment is included in the measure, ranking it 7389th (at 96.00 percentile) when using the measure *I*_total_, still a significant ability to retain structural information but not as impressive as the backbone-only case.

**Table 3:** Mutual information ranking for the prebiotic reduced alphabet

Interaction domain	Fold class [SCOP #]	Unweighted mutual information (*I*)		Normalized mutual information (*I*_norm_)	
		${\mathbf{\mathfrak{R}}}_{\mathrm{prebiotic}}^{\mathbf{10}}$ rank	${\mathfrak{R}}_{\mathrm{prebiotic}}^{10}$ percentile rank (%)	${\mathbf{\mathfrak{R}}}_{\mathrm{prebiotic}}^{\mathbf{10}}$ rank	${\mathfrak{R}}_{\mathrm{prebiotic}}^{10}$ percentile rank (%)
backbone (*I*_bb_)	all data [1–4, 7]	48	99.97	859	99.54
	all-α [1]	3351	98.19	1688	99.09
	all-β [2]	2834	98.47	1899	98.97
	α/β [3]	13	99.99	124	99.93
	α + β [4]	52	99.97	396	99.79
	small [7]	270	99.85	878	99.52
backbone + contact (*I*_total_)	all data [1–4, 7]	7389	96.00		
	all-α [1]	18,289	90.10		
	all-β [2]	18,333	90.08		
	α/β [3]	4127	97.77		
	α + β [4]	5325	97.12		
	small [7]	104,252	43.57		

The table shows the rank of the prebiotic reduced alphabet, in terms of mutual information (for both *I*_bb_ and *I*_total_), among the spectrum of all 184,756 possible 10-amino-acid reduced alphabets. The unweighted mutual information is described in Eq. (7) for *I*_bb_ and in Eq. ((2) for *I*_total_, while the normalized mutual information *I*_bb,norm_ is described in Eq. (8)

Distinguishing among the different fold classes as defined by SCOP, we observe, using the unweighted measure *I*_bb_, that the prebiotic set covers the backbone space of α/β, α + β, and small proteins impressively well, ranking 13th, 52nd, 270th over-all (at 99.99, 99.97, and 99.85 percentiles) respectively, while the all-α and all-β proteins are covered moderately well, ranking at 3351st and 2834th (98.19 and 98.47 percentile) respectively. The normalized measure *I*_bb,norm_ bears the same general patterns in ranking but with less variation, ranking the prebiotic set at 124th, 396th, and 878th (99.93, 99.79, and 99.52 percentiles) for α/β, α + β, and small proteins respectively, and 1688th and 1899th (99.09 and 98.97 percentiles) for all-α and all-β proteins respectively. The comparable outcomes for *I*_bb_ and *I*_bb,norm_ indicate that variation in amino acid frequency has a minimal effect, if any, on our results.

When the full structural information *I*_total_ is used to evaluate the behavior of the prebiotic set in different fold classes, a different pattern emerges. For α/β and α + β proteins, the prebiotic set retains the ability to preserve structural information, ranking it 4127th and 5325th (97.77 and 97.12 percentiles) respectively. However, for all-α and all-β proteins, a significant degradation is observed, pushing the ranking of the prebiotic set to 18,289th and 18,333rd (90.10 and 90.08 percentiles) respectively. For small proteins, the prebiotic set lands on its worst ranking of all, at 104,252nd (43.57 percentile).

From these measurements, the following key observations can be made. First, the prebiotic set is superior in covering the backbone structural space inhabited by extant proteins. Second, when the necessary tertiary contacts in the context of the folded proteins are considered, the ability of the prebiotic set to retain structural information depends greatly on the fold class. The prebiotic set has near-optimal coverage of the structural space of α/β proteins, and to a slightly lesser extent, α + β proteins. This is true when the backbone structure is considered exclusively (both unweighted and sequence-normalized measures) or when the residue contact environment is considered. Third, the prebiotic set performs least optimally in preserving fold information in all-α and all-β proteins, particularly when the tertiary interactions present in folded proteins are considered. Fourth, while the backbone space for small proteins is significantly covered by the prebiotic set, their residue contact environments require significantly different reduced alphabets from the prebiotic set.

### Gapless threading by random sequence shuffling

Natural proteins built from the full genetically-coded amino acid alphabet have the crucial ability to distinguish correct folded conformations from all other incorrect folds, by virtue of the prominent energy gap that characterizes the energy landscape. Gapless threading was done to assess the ability of the reduced prebiotic set to maintain such discrimination. Results of the threading simulation, summarized in Table 4, show that, for threading using the backbone potential only, there are only 6 reduced alphabets (out of 184,756, or 99.997%) that are better than the prebiotic set at discriminating native sequence-structure alignment from incorrect alignments. For threading using the total potential, which incorporates non-local interactions, there are 1858 10-set alphabets that are better than the prebiotic set (99.00%), still significant but not extraordinarily so.

**Table 4:** Gapless threading results

Fold class [SCOP #]	${\mathfrak{R}}_{\mathrm{prebiotic}}^{\mathbf{10}}$			${\mathfrak{R}}_{\mathrm{best}}^{\mathbf{10}}$			Rank of ${\mathfrak{R}}_{\mathrm{prebiotic}}^{\mathbf{10}}$ (out of 184,756)
	Δ*U*_*native*_/*kT*	<Δ*U*_*incorrect*_> */kT*	Δ*U*_*native*_/*kT* - <Δ*U*_*incorrect*_> */kT*	Δ*U*_*native*_/*kT*	<Δ*U*_*incorrect*_> */kT*	Δ*U*_*native*_/*kT* - <Δ*U*_*incorrect*_> */kT*	
*backbone interaction domain* (*I*_bb_)							
All data [1–4, 7]	− 0.2161	0.3590	− 0.5751	− 0.2171	0.3609	− 0.5781	7
α only [1]	− 0.4649	0.2909	− 0.7558	− 0.4647	0.4260	− 0.8907	451
β only [2]	− 0.1014	0.3363	− 0.4377	− 0.1134	0.3983	− 0.5116	455
α/β only [3]	− 0.2453	0.3497	−0.5951	− 0.2482	0.3665	− 0.6148	3
α + β only [4]	− 0.2139	0.3419	− 0.5558	− 0.2154	0.3667	− 0.5821	31
small only [7]	0.0175	0.3503	−0.3328	0.0420	0.4493	−0.4073	4
*backbone + contact interaction domain* (*I*_total_)							
All data [1–4, 7]	−0.3429	0.6667	− 1.0096	− 0.3676	0.7367	−1.1043	1858
α only [1]	− 1.1000	0.5849	− 1.6848	− 1.1703	0.7039	−1.8743	3260
β only [2]	0.1886	0.7320	−0.5434	0.1724	0.8348	−0.6624	1013
α/β only [3]	− 0.2961	0.6558	− 0.9519	−0.3251	0.7188	− 1.0439	526
α + β only [4]	− 0.2240	0.6939	− 0.9180	− 0.2384	0.7876	− 1.0260	328
small only [7]	0.9708	0.7631	0.2077	0.6454	0.7844	− 0.1390	10,112

This table shows computed values of the knowledge-based potential defined in Eq. (12) and the fitness rank of the prebiotic set. The quantity Δ*U*_*native*_ refers to the free energy of the reduced sequence in its correct tertiary structure. The quantity <Δ*U*_*incorrect*_ > refers to the mean free energy of the shuffled reduced sequence mounted on the same tertiary structure. The difference between these two quantities measures the discriminative ability of the reduced sequence for its correct conformation. The substitution rules for both the prebiotic set and the best reduced set are outlined in Table 2. The rank of the prebiotic set is measured in terms of this discriminative ability, comparing this value to those given by the spectrum of 184,756 alternative reduced alphabets

Threading results for proteins partitioned by SCOP fold class reflect general observations seen in the mutual information measurements. Reduced sequences created from the prebiotic set are able to discriminate native-like conformations from incorrect folds for α/β and α + β folds better than for all-α and all-β proteins. Considering only the backbone structure space, only 2 and 30 other reduced alphabets do better in threading discrimination than the prebiotic set for α/β and α + β proteins respectively. When long-range interactions are taken into account, the discrimination of the prebiotic set in α/β and α + β folds is superior to all but 525 (99.34 percentile) and 327 (99.82 percentile) of all other reduced alphabets respectively. The prebiotic set performs the worst in discriminating the native conformation of small proteins against incorrect folds, ranking only 10,112nd (94.53 percentile) compared to alternative alphabets.

## Discussion

Explorations of the creation of the first living cells on Earth, born from early organic molecules that catalyzed reactions for self-propagation and metabolism, can be aided by studies of the biochemistry of current living organisms. Since it is possible that the ingredients that built these prebiotic organic molecules are similar to what composes extant biosystems, we may be able to learn about primordial evolution from our current biochemical inventory [44]. Proteins are such an integral part of every aspect of life that the story of how life began, persisted, and flourished must necessarily account for the emergence and propagation of peptides in the evolutionary timeline. Extant proteins should provide a rich catalog of molecular fossils that potentially bear sequence and structural artifacts dating back to the earliest functional peptides.

Studies have posited a temporal division in the emergence of the twenty genetically-coded amino acids, identifying a subset as prebiotic, likely existing before cellular life began, and possibly participating in the formation of the earliest functional peptides [2, 5, 6]. Most conclude that 10–12 amino acids, including {A,D,E,G,I,L,P,S,T,V}, appeared early, with the rest gradually added to the repertoire as their anabolic pathways became established [1, 8]. Some have even postulated a specific order of emergence for these amino acids [45–47].

A fundamental question is whether polypeptide chains formed predominantly from the set of prebiotic amino acids were sufficient to formulate functional molecules to begin and sustain the first life. Though we may not know the full repertoire of functions that arose then, we can surmise that some of the functions of modern proteins must have originated from these primordial forms which have endured through cycles of advantageous modifications. Similarly, because there is a limited number of folds today compared to the diversity of natural sequences [22, 23], useful folds are hypothesized to have persisted from the moment they arose in the timeline of molecular evolution [24], such that the set of extant protein structures has arisen from accretion and must include traces of the earliest folds. Thus, a way to explore the viability of the prebiotic amino acids in giving rise to functional folds is to examine their ability to preserve structural information and code for extant folds despite drastic alphabet reduction.

In line with these propositions, experimental studies have aimed to simplify the amino acid sequence of particular proteins without altering structural and/or functional integrity [12–18, 48, 49]. These demonstrations provide a compelling proof of concept that reduced alphabets, including the prebiotic set, can formulate functional folds. In this study, we sought to know if this growing experimental evidence can be generalized across diverse sequences and folds by examining whether these patterns stem from fundamental structural properties of optimized reduced sets and of the prebiotic amino acid set in particular.

To explore the structural properties of reduced alphabets, we designed a way to quantify the ability of any alphabet to retain sufficient structural information to preserve folds, by applying virtual mutagenesis to single-domain protein sequences of diverse conformations. Two principal measures of structural information were computed—one is the ability of the local sequence to encode backbone structure, and the other includes the ability of the contacting residues far away in sequence to encode the folded molecular environment. The former, *I*_bb_, chiefly measures the local backbone propensities of sequences, but since the mutual information is parameterized using X-ray crystal structure data of folded proteins, this quantity more accurately characterizes backbone propensities of polypeptides as they are subjected to crowded conditions with many close but non-specific molecular interactions. Thus, it is appropriate to use *I*_bb_ to probe the functional fitness of the reduced alphabet in primordial conditions, where short peptides must have interacted fleetingly with and/or bound non-specifically to other molecules—including other peptides, nucleotides, mineral surfaces, metal ions, and other cofactors—in some density. The latter, *I*_total_, considers the full intramolecular structure of the folded sequence, specified by the backbone conformation and the matrix of non-local contacts that attend every residue position. This combined quantity effectively measures the ability of a reduced alphabet to formulate complete folds. Our information-theoretic approach allows us to systematically compare the fitness of the prebiotic set with all other possible 10-member alphabets, and consequently to detect whether the set of prebiotic amino acids exhibits unusual structural coding properties relevant to proteogenesis. We outline aspects of the prevailing story of molecular evolution below in light of the results of our work.

First, we can learn about the kinds of conformations that may have been generated prebiotically. Though short peptides do not exhibit conformational specificity because of the absence of a stabilizing tertiary framework, their backbones bear particular structural propensities [50, 51], depending significantly on the local sequence [52], as seen in the effect of the amino acid and its immediate neighbors on the phi-psi dihedral angles [31, 53–55]. These peptides likely aggregated non-specifically and also may have formed reasonably compact structures in interaction with other molecules and ions to form early functional conformations [56–62]. Compaction may have been necessary to avoid hydrolysis and may have facilitated the enrichment of soluble proto-enzymes [63]. While short amino acid sequences are not expected to condense into well-defined conformations, we can gauge the structural permissivity of sequences formed by reduced alphabets. The quantity *I*_bb_ measures the extent to which the reduced alphabet is able to formulate sequences with structural propensities resembling the backbone distribution of extant proteins as they are subjected to a statistical ensemble of nonlocal interactions in the molecular environment. The higher the *I*_bb_, the better the alphabet preserves the backbone structural information encoded in the full-alphabet sequence.

The extraordinarily high ranking of the prebiotic set based on *I*_bb_ shows useful characteristics of the polypeptides that would have arisen in early evolution. We observe that such prebiotic peptides optimally embody the backbone structural propensities of the extant universe of functional single-domain folds. Thus, if they were to formulate useful structures, they ought to have given rise to the kinds of local forms and surfaces that we observe in current proteins. These structures in principle could then participate in rudimentary biochemical catalysis.

Examination of *I*_total_ gives us a broader picture of the ability of reduced alphabets to preserve the structural information in full-alphabet sequences of extant proteins. We observe that the ranking of the prebiotic alphabet, while still in the 96th percentile, decreases markedly compared to its superior ranking for *I*_bb_. This suggests that the prebiotic set encodes functional backbone conformations optimally and functional tertiary conformations near-optimally. The prebiotic set alone is sufficient to code for the distribution of backbone conformations found in extant proteins, while a few of the later amino acids are needed to fully code for the observed range of tertiary conformations. These later amino acids appear significant to structure in the way they stabilize non-local tertiary interactions, implying that if the prebiotic set formulated nascent functional structures alone, they would have displayed marginal stability. This is significant because it is thought that folds of marginal stability, postulated to have arisen as life began, facilitated the evolution of diverse proteins of various functions [64–67]. Early transient structures were likely functionally promiscuous, accomplishing multiple roles in early metabolic pathways. Their marginal stability meant that, as life pushed towards more complex pathways, ancestral proteins more easily evolved with stepwise sequence mutations towards stable structures with more specific functions. Thus, tertiary conformations formed by early polypeptide sequences dominated by the prebiotic set provided a fertile ensemble of proto-enzymes that, in progressive molecular selection, could have generated the kinds of conformations that currently exist.

We then examined the fitness of the prebiotic set to encode backbone and tertiary conformations of individual SCOP fold classes. Using the rank of the mutual information given by the reduced alphabets, we find that the prebiotic set is most able to encode structures associated with the α/β fold, followed closely by the α + β fold. Structures found in folds consisting of all-β and all-α structures were less amenable to being conceived by the prebiotic set. The tertiary matrix of small proteins do not appear to be encoded by the prebiotic set at all. Thus, a proteomic system dominated by prebiotic amino acids would be expected to formulate mostly α/β and also some α + β folds, if any folds arise at all. Conversely, folds that contain all-α and all-β structures, along with small proteins, are the least likely to form. Examining the optimal 10-amino acid alphabets for the different fold classes (Table 2), we find that there is increasing dependence on the later (non-prebiotic) amino acids for the stabilization of conformations belonging to all-α, all-β, and small proteins. Therefore, if each of the 20 canonical amino acids actually emerged at different points in time, and if the prebiotic set approximately preceded the later ten, then our results hold that the α/β fold class emerged first in evolution, followed closely by α + β, and then later on the all-α fold class, all-β fold class, and small proteins. This timeline comports with other proteomic studies hypothesizing the order of proteogenesis [25–27, 68, 69]. The mixed α-β folds, not coincidentally, appear to dominate metabolic functions that would have been critical to the formation of early pathways [68, 70].

A number of bioinformatic studies have observed a shift in amino acid usage in proteins, consistent with the hypothesis that the prebiotic amino acids dominated ancestral sequences, with later amino acids entering the proteome successively [6, 71–75]. The optimal alphabets, based on the two measures *I*_bb_ and *I*_total_, adhere to the temporal order of the emergence of amino acids vis-à-vis that of the emergence of different folds. Trifonov [45] hypothesized the following order for the later 10 amino acids, based on a synthesis of multiple genomic and proteomic criteria and hypotheses: R/(Q,N)/H/K/C/F/Y/M/W; Sobolevsky & Trifonov [46], in an analysis of octapeptides in bacterial proteomes, produced the following order: R/Q/N/K/F/H/C/M/Y/W; and Liu et al. [47], from a statistical analysis of amino acid usage across species, proposed the following order: K/R/N/F/Q/Y/M/H/W/C. According to our findings pertaining to *I*_bb_ optimization, in order to encode the backbone conformations of all-α and all-β proteins optimally, amino acids towards the beginning of the three temporal orders—N, K, Q, and H—in addition to the prebiotic set, appear to play significant roles. According to *I*_total_ optimization, however, the amino acids towards the end of the order—C, F, Y, and W—become significant for tertiary stability. Cysteine is a creative addition to the protein building block for its ability to form disulfide bonds, while the aromatics phenylalanine and tryptophan scaffold a highly hydrophobic core. A deeper analysis of such amino acid reduction and substitution patterns is necessary to discern the roles each amino acid takes in the emergence of stable functional structures along the evolutionary timeline.

Finally, our work illuminates an important energetic aspect of sequences formed by the prebiotic amino acids. Modern proteins are said to be “minimally frustrated,” a characteristic referring to the consistently stabilizing additive interactions throughout the sequence, whose energy landscape features a pronounced gap between the spectrum of energies of the decoy ensemble and the energy of the native state [38]. Such an energy gap appears to be the result of energetically beneficial mutations that accrue, in the course of evolution, across the sequence. At which point in proteogenesis this property arose in ancestral proteins remains an open question. From our threading results, we observe that reduced sequences composed of prebiotic amino acids exhibit an exceptionally wide energy gap compared to all other alternative reduced alphabets. Thus, it appears that prebiotic sequences, especially those that form the earliest postulated folds α/β and α + β, are already amenable to mutations that increase the energy gap and smoothen the energy landscape, permitting efficient folding. It is noteworthy that this important folding property may have already been operative in ancient proteomes *prior* to the emergence of the full twenty genetically-coded amino acids.

## Conclusions

If the first polypeptides were constituted predominantly by prebiotic amino acids {A,D,E,G,I,L,P,S,T,V} on early Earth, then it would have been a significant advantage if this reduced alphabet bore some intrinsic propensity towards functional forms compared to other reduced alphabets. We investigate this conjecture by demonstrating the capacity of reduced sequences to form the kinds of structures that compose extant proteins, working from the assumption that ancestral proteins resembled proteins in our current proteomic inventory structurally and functionally. Our structure-based analysis probes the reducibility of the sequence space of all extant single-domain folds via a systematic virtual mutagenesis procedure. The methodology involves an efficient search across 10-member set reductions of the amino acid alphabet space (numbering more than 10^15^) in the virtual mutagenesis of more than 2000 single-domain proteins, and evaluating the fitness of each substitution using an optimized information-theoretic metric that encompasses both local backbone and long-range contact interactions.

We find that the prebiotic set is optimal in encoding the spectrum of local backbone structures that appears in the folded environment of extant proteins. This is relevant to the mechanics of proteogenesis in the following way. We imagine that prebiotic amino acids, which may have existed in significant concentrated quantities in some environments, formed short polypeptides that polymerized into longer chains and/or aggregated around cofactors, metals, nucleotides, other peptides, and other organic molecules. Our finding suggests that these prebiotic polypeptides, under crowded conditions that encourage close but non-specific interactions, are biased for backbone conformations embodied by modern folds. Thus, if any prebiotic polypeptide sequences formed structures that persisted long enough to be useful, they would have resembled conformations whose backbones are similar to extant proteins, and thus likely had functions that have persisted in current metabolic pathways.

We also find that the prebiotic set is near-optimal in encoding the spectrum of single-domain folds that exist in nature currently. There are fold-specific differences in the ability of the prebiotic set to encode the spectrum of tertiary conformations of extant proteins. The prebiotic set is able to encode native tertiary conformations of α/β and α + β folds, and, to a lesser extent, of all-α and all-β folds. The prebiotic set is insufficient to encode small proteins as a class, necessitating the stabilizing effect of later amino acids. These observations, based solely on structural criteria, comport with the consensus of a number of studies based principally on the analysis of proteomic and genomic sequences. First, regarding the timeline of first appearance of folds vis-à-vis the temporal order of amino acids, it makes sense that the earliest polypeptides dominated by prebiotic amino acids formulated simple α/β and α + β folds more readily than others. Since enzymes belonging to these folds are found in a wide range of metabolic functions, including the biosynthetic pathways of the later amino acids, it is likely that their emergence early in evolution facilitated the metabolic production and subsequent enrichment of later amino acids. Later amino acids would have entered the alphabet as their biosynthetic pathways were established by the ancestral folds. Second, suboptimal tertiary contacts make structures only marginally stable, allowing these proteins to be plastic and functionally promiscuous, an advantage early in evolution when the prebiotic proteome was small. The ancestral structures were also easily mutable and open to evolve other functions. With the expansion of the amino acid alphabet, conformational stability and functional specificity of later all-α and all-β proteins increased, and small proteins became possible, further widening the functional repertoire of enzymes.

Finally, we observe that sequences formulated exclusively by the prebiotic set are able to discriminate native over incorrect folds exceptionally well compared to all other possible reduced alphabets, particularly for those proteins belonging to the α/β and α + β fold classes. To have such a critical property of protein folding potentially arise early in proteogenesis, even prior to the establishment of the full 20 letter amino acid alphabet in the genetic code, would have facilitated the evolution of functional proteins with persistently stable structures, establishing a framework for the first metabolic pathways.

## Data Availability

All data generated or analyzed during this study are either described or included in this published article. Any other data that were derived by our methodology but not explicitly included in the current study are available from the corresponding author on reasonable request.

## Abbreviations

${\mathfrak{R}}^n$: *n-*member reduced alphabet, where 1 ≤ *n* ≤ 20; for instance, ${\mathfrak{R}}^{10}$ is a 10-member reduced alphabet; ${\mathfrak{R}}_{\mathrm{prebiotic}}^{10}$ is the prebiotic reduced alphabet made up of the ten amino acids {A,D,E,G,I,L,P,S,T,V}
$\mathcal{S}$: substitution rule, i.e. how amino acids not part of the reduced alphabet are to be mutated; ${\mathcal{S}}_{\mathrm{opt}}$ is the optimal substitution rule
*C*: protein conformation; *c* is a particular instance of conformation; *C*_c_ is non-local contact conformation; *C*_bb_ is the backbone conformation
*S*: amino acid sequence; *s* is a particular instance of sequence; *S*_loc_ is the local sequence
*I*_total_: total mutual information between sequence and conformation that considers backbone and non-local contact interactions
*I*_bb_: mutual information that considers only backbone interactions
*I*_c_: mutual information that considers only non-local contact interactions
α: weight coefficient in *I*_total_ that modulates the contribution of the two terms
(γ_i-1_,γ_i_): dihedral angle pair defined along the virtual alpha carbon C^α^_i-1_-C^α^_i_ bond (γ_i-1_) and the virtual alpha carbon C^α^_i_-C^α^_i + 1_ bond (γ_i_)
*p*: probability
*f*: frequency derived from the data set that is used to estimate *p*
*V*^*k*^(*γ*_*i* − 1_, *γ*_*i*_): Voronoi cell to which the dihedral angle pair (γ_i-1_,γ_i_) belongs
Δ*U*: change in potential
PISCES: Protein Sequence Culling Server, the source of our non-redundant data set
SCOP: Structural Classification of Proteins, used to classify proteins into fold classes
